# GPS Velocity and Strain Rate Fields in Southwest Anatolia from Repeated GPS Measurements

**DOI:** 10.3390/s90302017

**Published:** 2009-03-17

**Authors:** Saffet Erdoğan, Muhammed Şahin, İbrahim Tiryakioğlu, Engin Gülal, Ali Kazım Telli

**Affiliations:** 1 Afyon Kocatepe University, Geodesy and Photogrammetry Engineering Department, Afyonkarahisar, Turkey. E-Mails: itiryakioglu@aku.edu.tr; aktelli@aku.edu.tr; 2 İstanbul Technical University, Geodesy and Photogrammetry Engineering Department, İstanbul, Turkey; E-Mail: sahin@itu.edu.tr; 3 Yıldız Technical University, Geodesy and Photogrammetry Engineering Department, İstanbul, Turkey; E-Mail: egulal@yildiz.edu.tr

**Keywords:** Burdur-Fethiye fault zone, GPS, strain

## Abstract

Southwestern Turkey is a tectonically active area. To determine kinematics and strain distribution in this region, a GPS network of sixteen stations was established. We have used GPS velocity field data for southwest Anatolia from continuous measurements covering the period 2003 to 2006 to estimate current crustal deformation of this tectonically active region. GPS data were processed using GAMIT/GLOBK software and velocity and strain rate fields were estimated in the study area. The measurements showed velocities of 15–30 mm/yr toward the southwest and strain values up to 0.28–8.23×10^−8^. Results showed that extension has been determined in the Burdur-Isparta region. In this study, all of strain data reveal an extensional neotectonic regime through the northeast edge of the Isparta Angle despite the previously reported compressional neotectonic regime. Meanwhile, results showed some small differences relatively with the 2006 model of Reilinger *et al*. As a result, active tectonic movements, in agreement with earthquake fault plane solutions showed important activity.

## 1. Introduction

Anatolia is one of the most seismically active regions of the Mediterranean basin. Historical seismicity, as well as instrumental recordings of earthquakes occurred in the last 10 years, reveals the active tectonic features of this area. The North Anatolian, East Anatolian, Aegean, and Burdur-Fethiye fault zones, point to the tectonic diversity and activity of Anatolia. Since the mid 1980s, the Global Positioning System (GPS) has provided earth scientists with new opportunities to estimate present-day surface kinematics and Earth’s crust deformations [1,2]. In the eastern Mediterranean, previous GPS studies have helped quantify large-scale plate motions [3,4], regional deformation in the zone of plate interaction [5–7], and deformations associated with the earthquake cycle [8]. GPS studies conducted in recent years have demonstrated the presence of a recent NE-SW extension in Western Anatolia, which moves at about 30 mm/year towards the southwest [7].

During the last 40 years, the region has been extensively studied and mapped and recent publications show the paleotectonic structure of the region. However, active crustal extension has never been observed in the regions of Isparta and south of Burdur [9]. Barka *et al*. [10] indicated that velocity vectors located in the vicinity of the Burdur-Fethiye Fault zone are oblique to main trend of the fault zone. The slip vector obtained from inversion solution of the Dinar earthquake is nearly parallel to that obtained from GPS surveys. This direction of the slip vector is expected to cause normal faulting along the Dinar Fault with slight right-lateral component, and combination of left-lateral and extension along the Burdur-Fethiye Fault zone. The 1971 Burdur earthquake indicated pure normal faulting parallel to the Burdur-Fethiye Fault zone [10]. Although the region has been studied and mapped, any specific model related to the region could not be concluded. The aim of this study is to reveal the updated kinematics related with strain rates, and deformations of the region.

## 2. Burdur-Fethiye fault zone

**Figure 1.**
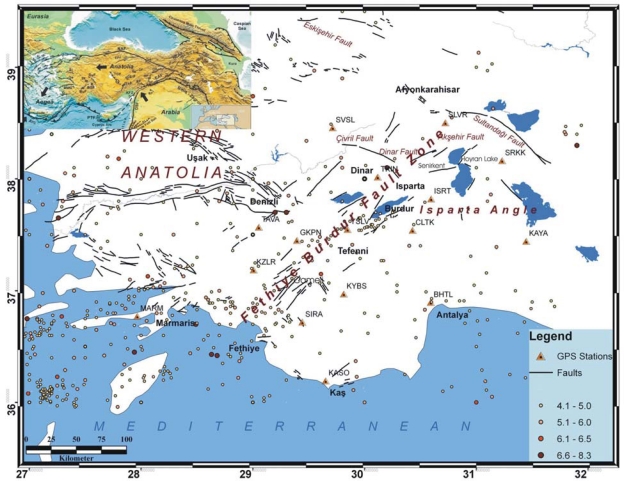
Outline tectonic map of the easternmost Mediterranean area, showing the main neotectonic lineaments and the main structural units in the text. Seismicity of the Western Anatolia in the last century with *M*s > 4.

The Burdur-Fethiye fault zone is located in the southwest of Turkey in a tectonically active area. This fault zone, extending for 300 km between Burdur and Fethiye, is one of the most important zones and has produced many earthquakes in the recent past (Figure 1). The fault follows Senirkent and Hoyran Lake towards the northeast and intersects with the Akşehir-Simav faults in the Afyonkarahisar Çay region [11, 12, 13]. Meanwhile, the Burdur and Akşehir-Simav faults form fracture lines, which limit the geological structure known as the Isparta Angle [14]. The Burdur fault line is the most important fault in the region, and events from Fethiye Gulf to Hoyran Lake strike to the northeast as an en-echelon shaped system [15].

In most places, the fault zone is not one structural line but consists of short segments having discontinuous parallel lines. These segments are included in a zone, which lies towards the northeast, and has a width ranging between 3 and 10 km. Segments which extend to the northeast and are included in the zone are mostly limited by younger faults which are normal, direction pitched, and fully grown towards the north and northeast [16]. However, in the district located around the Çameli basin, the width of the tectonic zone reaches up to 30 km. Most of the faults located within the Burdur-Fethiye fault zone also carry the growth faults delimiting the neogene basins of the region [9]. The Cyprus and Hellenic Arc zones form an angle in the Fethiye Gulf shoreline. Formations of this angle have a geometric relationship with the Burdur fault’s slip, which is to the left (Figure 1).

GPS studies of the area, including the Isparta Angle, were performed, and these studies showed that movements in the Isparta Angle are 10 mm less than the movements in the Eurasian plate [17]. The Isparta Angle is a triangular-shaped structure about 120 km long N-S and 50 km wide in the south, extending offshore into the Antalya basin [18]. Southwestern Anatolia, which is delimited by the Burdur-Fethiye fault zone and associated with the Isparta Angle, is rather under the influence of a SW-NE directed compressive regime unlike the Aegean region. Therefore, the Fethiye-Burdur fault zone constitutes a very critical structural line separating two different tectonic zones in Western Anatolia [9].

**Figure 2.**
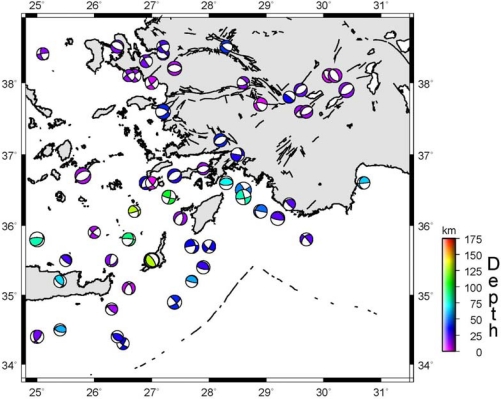
The focal mechanisms of the earthquakes in the Southwest Anatolia (M > 5.5) [19].

Three main earthquakes of magnitudes ranging between 6.1 and 7.1 occurred in the region in 1914, 1957, and 1971 in the last century. Figure 2 illustrates the focal mechanisms of the earthquakes (M>5.5) that took place in SW Turkey and in the vicinity of NE Hellenic Arc region. Most of the fault plane solutions of the events since 1977 are taken from Harvard Centroid Moment Tensor (CMT) catalogue. Also, the focal mechanisms of the 1914 Burdur earthquake (M=6.9) and the focal mechanisms of the 1957 Fethiye-Rhodos earthquake (M=7.1) are included. These are the largest two events to have affected the study region [19] (Figure 1).

## 3. GPS Observation and Data Processing

The study area occupies approximately 45.520 km^2^. The GPS network consists of ten pillar points and six bronze benchmarks in the rock. The GPS sites used in the network were considered to be outside of the fault zones, and thus not subject to plastic or elastic deformation. The longest base line SRKK-MARM has a length of 328 km. The shortest baseline is ISRT-CLTK with a length of 35 km. To determine the current kinematics of the region, five GPS surveys were performed during the 2003–2006 period using Ashtech and Thales GPS sensors. Two of them were conducted for a period of six months, with daily 12 hour sessions for each site. In the other three surveys, the network was extended with six new points and measurements were conducted over three days with nine-hour sessions and 15 sec sampling rate with a cut-off angle of elevation of 5°. TKIN and KYBS stations were used as permanent sites in the first four campaigns and continuous measurements were made. In the last campaign, KYBS changed to BHTL because of the logistic problems.

**Figure 3.**
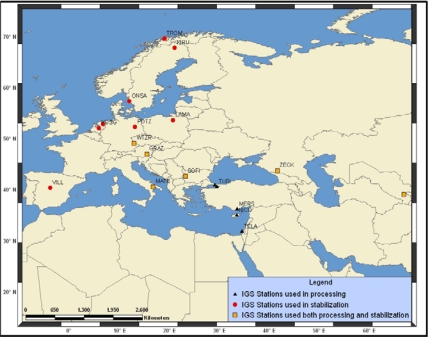
IGS stations used both processing and data transformation.

Data analysis was performed by using the GAMIT/GLOBK software. Raw data were converted into the RINEX format, using Ashtech Office Suite software. Evaluations were performed in three stages [17, 20]. In the first, a linear combination (LC) was formed for each day with GAMIT software. Using the formed LC station coordinates, atmospheric delays for each station, and orbit information, and input values for analysis were obtained without giving weight to the parameters. In this stage of relating the local network to the IGS network using coordinates which are mm. sensitive for calculating orbit information and earth rotation parameters, more accurate station points with respect to IGS networks were evaluated separately (Figure 3).

In the second stage, site coordinates, satellite coordinates, and covariance matrices were analyzed by Kalman filtering, without forcing, to obtain accurate velocities and coordinates. To provide stabilization during this stage, local solutions and global IGS solutions edited by SOPAC were evaluated, as well [7]. After stabilizing reference frame in the third stage, a reference system (European frame) was defined and velocity estimations were resolved to a European fixed reference frame that facilitates assessing the local deformation. Different site combinations were used for reference system definition. Horizontal velocities given by ITRF00 a-priori coordinates were minimized, and a Eurasian reference system was defined [7].

Many datum stabilization attempts have been realized by means of different site selections related to the region. Selection of sites for reference frame definition of both coordinates and velocities was made based on site history, long-term repeatability, geographic coverage of the region and availability in pre-processed regional solutions. Selected groups of sites according to criteria mentioned above are those defined as stable by Altamimi *et al*. [21], McClusky *et al*. [7], and used by those studies for reference frame definition. Site sets were formatted by different attempts and by extracting inappropriate sites (Figure 3). As a result, transformation parameters were obtained by generalized constraints to the point sets.

For a Eurasia-fixed frame, residual velocities of 14 IGS sites were obtained by differentiating ITRF2000 and Eurasia plate velocities. Post-fit RMS of 14 stations was found to be 2.7 mm and 0.4 mm/year for coordinates and velocities, respectively. Measurements performed as periods, were evaluated using daily measures for each. Normalized root mean square (nrms) and weighted root mean square (wrms) values were computed. Sites used for the reference frame and project sites definition were taken into consideration. Normalized root mean square (nrms), weighted root mean square (wrms) values, and daily repeatabilities were used to detect the errors caused by centralizing, and to define the quality of results.

**Figure 4.**
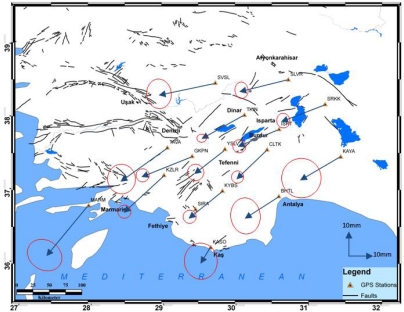
Horizontal velocity field in the Eurasia-fixed frame (ellipses are at 95 % confidence level).

Normalized root mean square (nrms) values exist in GAMIT q files, which include results related to daily solutions, are expected in the range of 0.15 and 0.25 for corresponding days for each period. Larger values bigger than the value of 0.5, indicate model errors or unblocked cycle clips. The site of SIRA was completely excluded because error ellipses of the velocity vector for this site were above the acceptable repeatability values. Before estimating velocities in the other step of our analysis, we examined the time series of position estimates to determine the appropriate weights to be applied to each period surveys (see Appendix). In a later stage, velocity values for different combinations were obtained using five periods’ measurements (Figure 4, Table 1).

**Figure 5.**
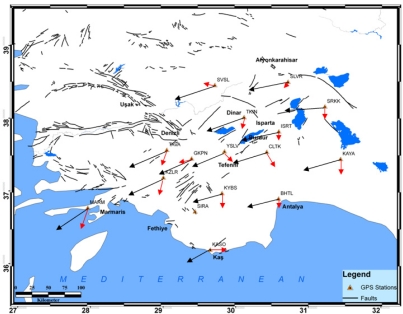
Residual values between measured horizontal values (red arrow) and Euler pole values of McClusky (black arrow).

McClusky *et al*. [7], as a result of the GPS surveys which they conducted for 189 points within a nine-year period between 1988 and 1997, calculated the Euler pole rotation values for the Eurasian/Anatolian tectonic plate as 30° 7′ latitude, 32° 6′ longitude, and 1.2 °/Ma. Employing these data, velocity values were computed for the points located on the Burdur-Fethiye fault zone. In the subsequent stage, local deformations were determined from the differences between the velocities of these points and those calculated on the basis of Euler values. To understand the differences in the movements of the region, the residual values of the Euler velocity vector values are shown in Figure 5.

## 4. Strain Analysis

The GPS data were collected and processed in order to provide station coordinates within a few millimeters’ accuracy. Displacements or velocities obtained by GPS data processing over repeated surveys can provide useful information on tensional states of terrestrial crust, in those areas in which many stations well spatially distributed are present [22]. In this study, strain rates were calculated from the GPS velocity field by grid_strain software [23]. The problem was considered as two-dimensional. Because GPS surveys can only provide deformation rates on the earth’s surface (x (EW) and y (NS) directions), and do not give any information on deformation rates in the radial direction (z). The grid_strain software automatically computes the grid length based on station baselines (in general, on spacing of the experimental points). The standard deviation of all the inter-distances between point pairs is computed and proposed as default value [23]. Once the grid is corrected based on user preferences, the program automatically define the smoothing parameter, or scale factor, for the modification of the least square weighting matrix as in Shen *et al*. [24].

The scale factors characterize the strain calculation. In particular, if data points are widely dense distributed, the local strain can be estimated at each node of the grid using a weighting strategy to automatically lower contribution of far stations from the node [23]. All data are involved in computation, but errors are rescaled using an appropriate function, which increases with distance. Following Shen and Jackson [25], the weigh function *e*−*d* / *d* 0 is used, where *d0* is the smoothing parameter, and *d* is the distance between the node and the grid point. Except for the weighting function, which is introduced a factor in the weight matrix considered in the least square computations, the approach is the same described in Livieratos [26].

**Figure 6.**
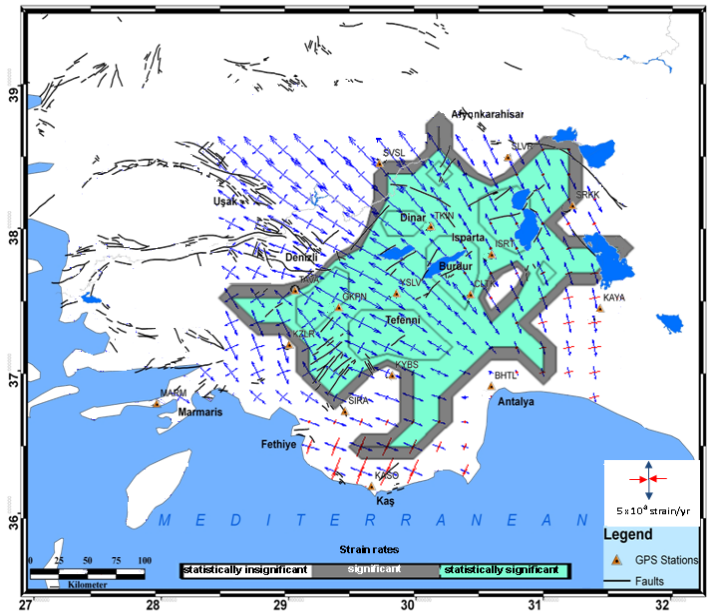
Principal Strain rates. Blue and red arrows show maximum extension and compression, respectively in 10 ^−7^ strain/year.

Once the grid (generated in a previous program session) and the scale factor are available, the program starts the strain rate estimation over each node of the grid. The computational function to solve for redundant equation system and estimate the velocity gradient tensor follows the standard linear least square approach but the weight function is introduced to error scaling. The results of the strain computation on all the grid points are presented immediately after the conclusion of the iterations [23]. The eigenvectors are presented with their directions and two colors: if an eigenvalue is positive, (extension) the color is blue, whereas; the color is red in the case in which the eigenvalue is negative (compression). Figure 6 and Table 2 present these results.

## 5. Results and Discussion

In this study, the current kinematic field of crustal motion, based on repeated GPS observations, is analyzed in terms of strain rates. From these results, it was found that the region is affected by a southwestern movement of 15–30 mm/yr. The most important findings of the study were velocity values for three years’ measurements of the region. Measurements, which were performed as periods, were evaluated using daily measures for each. Normalized root mean square (nrms) values existed in q files, which included results for daily solutions, and ranged between 0.17 and 0.24 for related days in each period. Average nrms values corresponding to periods are shown in Table 3.

The TAVA, SVSL, MARM, KASO, BHTK, and KAYA stations show larger error ellipses than other sites. The main reason for this was that velocity values for these sites were calculated from three campaigns in two years, while the other sites velocity values were calculated from five campaigns in three years. Another reason was that measurement durations were long and repeated measurements were not made during the first and second periods. Errors due to tool centralizing in repeated measurements caused greater error ellipses [26].

Velocities at the SLVR and SRKK stations are consistent with movements of the region. When strain values were investigated there was found to be an extension in the region. The strain vectors between the SLVR and SRKK points confirmed this extension. Furthermore, when the fault plane solutions of the earthquake of Sultandağı were considered, this extension structure is consistent with the velocity values too [27]. An examination of the ISRT, TKIN, and SVSL points revealed that the velocities were consistent with the tectonic features of this region, and that the extension at SLVR and SRKK points was observed here as well. NW-SE extension is also active between TKIN and SVSL stations. The cause of the expansion can be attributed to the Çivril fault. When the fault plane solutions of the earthquake of Dinar (1995) are examined (Figure 2) an NW-SE extension is shown contrary to the strain values of the region. The study showed that there was a strike-slip movement with a velocity of 3 mm/year between the YSLV and CLTK points, and this suggests that these points are located on the same zone, rather than being opposite to each other. In fact, these two points are situated on the same block in the model of Reilinger *et al*. [2]. The extension at the CLTK and TKIN points was quite apparent. Similarly, the opening between GKPN and YSLV demonstrated that these two points are located on different blocks. The extension structure obtained from the velocity values in the region of Burdur was consistent with the fault plane solutions of the earthquakes of Burdur (1914 and 1971).

An extension movement was observed between the CLTK and TKIN points, which have been defined by Yağmurlu *et al.* [9]. The reason for this fact is the periodic formation of NW-SE directed attraction forces and the related formation of NE-SW oriented normal faults in the zone around Lake Burdur, since the Yeşilova peridotite mass to the south of this lake is of a nature that partially restrains the SW-directed slipping movement. As for the GKPN-KYBS and GKPN-YSLV points, an extension movement was visible.

Between the TAVA and GKPN points, a slight compression in the NW-SE direction was observed. The NE-SW directed opening between the KZLR-TAVA and the KZLR-GKPN points suggest that the strike-slip moving the GKPN point is not located on the same block as the TAVA and KZLR points according to the model of Reilinger *et al*. [2]. Hence, it is assumed that either the boundary here must be stretching under the GKPN point or strike-slip faults are extremely active in the region. In this study, all of strain data reveal an extensional neotectonic regime through the northeast edge of the Isparta Angle despite the previously reported compressional neotectonic regime. An extension movement was observed between the CLTK and ISRT points, which has been defined by Yağmurlu *et al.* [14] as the “Antalya Fault Zone”, and by Glover and Robertson [28] as the “Kemer linearity”. The expansion between the Antalya and Isparta shows that the fault system which parallel to the Eğirdir Kovada graben is active [28].

Again, relative movement in the MARM point supports extension in the block models of Reilinger *et al*. [2] and others. Consideration of the KAYA site demonstrates that directional pitched movements are effective in the region, and the Kirkkavak fault, which is right directional, is verified. Consideration of the SRKK, SLVR, YSLV, KYBS, KASO, and BHTL sites, which are shown in the same block by Reilinger *et al*. [2], and lie on the right-hand side of the Burdur fault zone, shows that these sites have a counterclockwise rotation. When the fault plane solution of the earthquake of 1957 Fethiye-Kaş earthquake examined, it was consistent with the compression in the Kaş region. This supports the movement, which was highlighted but was not noted clearly by McClusky *et al*. [7, 29].

## 6. Conclusions

The principal findings of this study are GPS strain fields and velocity fields for the period 2003–2006 in the southwest of Turkey. Generally, the velocity field indicates counterclockwise rotation for the study area according to a Eurasia-fixed stabilization. Strain vectors show clockwise rotation for the study area. The velocity field and strain fields are characterized by a system that indicates non-deforming and deforming regions separated by fault zones. In addition, the velocity field is in agreement with location of seismicity, earthquake focal mechanisms, and mapped fault locations, as supported by former studies [30,2,7]. The southern part of the area shows slower movement than the north, according to GPS velocity vectors. The maximum velocity differences are up to 10 mm/yr between KYBS-KZLR and KASO-MARM stations. The strain values reach a maximum over the GKPN, SLVR, KZLR, SVSL and CLTK points.

## Figures and Tables

**Table 1.** Horizontal velocities and corresponding 1σ errors in Eurasia-fixed frame.

**Site**	**Lat (°)**	**Lon (°)**	**V_E_ (mm)**	**V_N_ (mm)**	**σ_VE_ (mm)**	**σ_VN_ (mm)**
**BHTL**	36.896	30.589	−13.45	−8.54	3.70	4.09
**CLTK**	37.53	30.427	−12.06	−11.45	1.47	1.55
**GKPN**	37.448	29.392	−20.15	−7.98	1.57	1.57
**ISRT**	37.82	30.592	−16.06	−6.92	1.51	1.61
**KASO**	36.194	29.648	−4.14	−5.95	3.97	4.24
**KAYA**	37.436	31.443	−15.9	−9.11	4.67	4.61
**KYBS**	36.971	29.81	−13.22	−11.14	1.52	1.47
**KZLR**	37.187	29.003	−15.92	−14.76	1.49	1.49
**MARM**	36.772	27.963	−16.55	−20	4.25	3.69
**SLVR**	38.503	30.72	−19.38	−4.82	1.69	1.90
**SRKK**	38.163	31.228	−16.91	−6.77	1.56	1.74
**SVSL**	38.458	29.711	−22.40	−4.67	3.10	3.36
**TAVA**	37.566	29.048	−18.13	−13.42	3.36	3.66
**TKIN**	38.016	30.114	−16.79	−8.93	1.13	1.01
**YSLV**	37.547	29.844	−11.43	−9.34	2.15	2.25

**Table 2.** Principal strains computed from GPS velocities

**Site**	**Lon (°)**	**Lat (°)**	**ε_max_ (10^−7^ year^−1^)**	**σε_max_ (10^−7^ year^−1^)**	**ε_min_ (10^−7^ year^−1^)**	**σε_min_ (10^−7^ year^−1^)**
**BHTL**	36.896	30.589	0.051	0.184	−0.027	0.046
**CLTK**	37.530	30.427	0.691	0.048	0.015	0.279
**GKPN**	37.448	29.392	0.804	0.073	0.198	0.421
**ISRT**	37.820	30.592	0.507	0.061	0.022	0.193
**KASO**	36.194	29.648	0.368	0.297	−0.601	0.870
**KAYA**	37.436	31.443	0.333	0.233	−0.203	0.699
**KYBS**	36.971	29.810	0.496	0.072	0.130	0.200
**KZLR**	37.187	29.003	0.541	0.474	0.236	1.407
**MARM**	36.772	27.963	0.386	0.027	0.159	0.271
**SLVR**	38.503	30.720	0.585	0.030	0.003	0.093
**SRKK**	38.163	31.228	0.538	0.039	−0.045	0.099
**SVSL**	38.458	29.711	0.868	0.026	0.087	0.155
**TAVA**	37.566	29.048	0.554	0.071	0.250	0.463
**TKIN**	38.016	30.114	0.428	0.039	0.023	0.203
**YSLV**	37.547	29.844	0.723	0.062	0.099	0.323

**Table 3.** Average nrms and wrms values with related periods in project sites and IGS sites.

	**IGS SITES**				**PROJECT SITES**			
	**NORTH**		**EAST**		**NORTH**		**EAST**	
	**nrms**	**wrms**	**nrms**	**wrms**	**nrms**	**wrms**	**nrms**	**wrms**
**2003**	0.4	0.3	0.6	0.6	1.9	2.7	2.0	3.2
**2004**	0.5	0.4	1.0	1.4	0.2	0.2	1.5	2.4
**2004/2**	3.9	3.7	3.0	3.9	2.7	6.1	2.4	6.1
**2005**	4.8	13.2	2.0	7.7	3.1	16.5	2.7	17.7
**2006**	1.3	1.5	1.3	1.8	1.8	3.6	2.0	5.9
